# Standardizing Training in Mindfulness-Based Interventions in Canadian Psychiatry Postgraduate Programs: A Competency-Based Framework

**DOI:** 10.1007/s40596-017-0721-5

**Published:** 2017-06-21

**Authors:** Andrea Grabovac, Erin Burrell

**Affiliations:** 1 University of British Columbia, Vancouver, British Columbia Canada; 2 University of British Columbia, Victoria, British Columbia Canada

The Association of Medical Faculties of Canada has set a strategic direction to transform medical education through the implementation of competency-based training programs [1]. We propose an application of this paradigm to the training of Mindfulness-Based Interventions (MBIs) in Canadian psychiatry postgraduate programs. We have developed competency-based guidelines, informed by a survey of postgraduate directors on current MBI training opportunities across Canada, as well as an examination of the unique training requirements associated with MBIs.

MBIs such as Mindfulness-Based Stress Reduction (MBSR), Mindfulness-Based Cognitive Therapy (MBCT), and Mindfulness-integrated Cognitive Behaviour Therapy (MiCBT) are manualized individual and/or group treatments that emphasize mindfulness practices, along with psychoeducation and reflective discussions, termed inquiry [2–4]. MBIs have proliferated and are now used to treat a broad range of psychiatric disorders, including depressive and anxiety disorders, substance use disorders, eating disorders, and insomnia [5–9]. MBCT is one of the most researched and applied MBI adaptations within psychiatric practice. It has level 1 evidence as a first-line maintenance treatment for major depressive disorder and is as effective as remaining on maintenance antidepressants, with no significant difference in cost [10, 11].

The current Objectives in Training in psychiatry for the Royal College of Physicians and Surgeons of Canada requires that graduates have “introductory knowledge in assessing suitability for, prescribing, and delivering … mindfulness training” [12]. It is therefore incumbent on residency programs to provide introductory MBI instruction so that all residents are qualified to assess patient suitability for MBIs, as well as to prescribe these treatments. We argue that “introductory knowledge in delivering” MBIs as described in the Objectives of Training is not adequate for safe and competent MBI treatment delivery. Furthermore, it should not be expected that all psychiatrists have competency in MBI delivery. Rather, only those clinicians who elect to provide mindfulness-based psychotherapies require advanced training.

Although general training standards, certification programs, and assessment criteria for MBI clinicians have been proposed, we are unaware of training guidelines or defined competencies for the training of psychiatrists and psychiatric residents in MBIs, such as MBCT, directly serving psychiatric populations [13–17]. Standardized competencies within psychiatry have the potential to promote targeted learning and clear assessment standards, and complement the Competence by Design framework developed by the Royal College of Physicians and Surgeons of Canada, which represents a transformational change in postgraduate training and is scheduled for implementation between 2018 and 2022 [18].

The Competence by Design framework emphasizes outcomes-driven education and assessment across the spectrum of medical training, in contrast to the current emphasis on defined time periods within rotations [18]. Knowledge and abilities are specified for six stages of practice, two of which are applicable to MBI training: Core of Discipline (Core), which defines abilities integral to all psychiatric practice, and Advanced Expertise (AE), which denotes development of specialized skill sets [18]. The Competence by Design framework is structured around Entrustable Professional Activities (EPAs), which refer to tasks in the clinical setting that a supervisor can delegate to a resident, once sufficient competence has been demonstrated. Organized by CanMEDS roles, discrete skills and attitudes required for competency with a specific EPA are termed “milestones” and serve as observable markers of an individual’s ability at a specific stage of expertise [18, 19]. EPAs can be used as assessment tools to ensure that relevant milestones have been learned, and the milestones themselves can be used to design curricula and implement targeted teaching.

We define the EPA of assessing patients for suitability and appropriately prescribing MBIs as a Core of Discipline activity, for which all psychiatry trainees should be equipped. Some clinicians will also develop specialization in delivering MBI treatments and, for senior psychiatrists, in providing MBI training and quality improvement leadership. To characterize these Advanced Expertise (AE)-stage skills, in the absence of a precedent within the Competence by Design framework, we have created two sub-stages: *AE-Therapist* and *AE-Leader*. Program directors can use the Core competencies to formalize program-wide training, while a subset of residents and practicing psychiatrists can be guided by the milestones associated with the AE-Therapist EPA to plan MBI electives and Continuing Professional Development opportunities.

In addition to defining EPAs and milestones, we propose MBI training pathways with examples of specific training experiences for each stage that can be integrated into psychiatry postgraduate training and Continuing Professional Development programs. Approaches to possible implementation challenges are also discussed.

## Proposed Canadian Psychiatry MBI Competencies

### Core Stage of Competency

Psychiatric populations are at increased risk for experiencing symptom exacerbation during MBIs, for example, the triggering of severe anxiety by maintaining attentional focus on the breath or body in the context of trauma history, or aggravating rumination during thought-focused practices [20]. Although most patients with anxiety and depressive disorders benefit from acquiring mindfulness skills, all psychiatrists must be equipped to assess suitability in the context of a patient’s unique illness, rendering this a Core stage EPA (Table 1a).

**Table 1:** Mindfulness training competency milestones by stage of training and by CanMEDS role

*(a) Core of discipline stage (all residents and psychiatrists)*
Entrustable Professional Activity (EPA): assess for suitability and prescribe an appropriate Mindfulness-Based Intervention (MBI)
*Medical expert*
• Describe the basic psychological framework underlying MBIs and how mechanisms developed through mindfulness practice result in symptom reduction
• Be aware of the evidence base regarding efficacy of MBIs in various clinical populations
• Exercise appropriate patient selection for specific MBIs based on indications, contraindications, and alternate treatment options
• Inform patients about expected risks and benefits in the context of best evidence and guidelines
• Address common misconceptions about MBIs and possible barriers to participation
• Recognize when personal values, biases, or perspectives may have an impact on assessment and influence either under- or over-prescription of MBIs
*Health advocate*
• Promote role of MBIs in self-management, relapse prevention, and maintaining wellness within and beyond the clinical environment
*(b) Advanced expertise–therapist stage (self-chosen residents and psychiatrists)*
Entrustable Professional Activity (EPA): deliver a manualized MBI to individuals or groups for whom it is indicated, with fidelity to core aspects of mindfulness-based teaching (assumes core milestones are met)
*Medical expert: Perform a patient-centred clinical assessment and establish a management plan*
• Devise an individualized formulation for each patient, establishing a rationale for selection of an MBI as a treatment of choice
• Demonstrate an awareness of psychological frameworks underlying MBIs
• Identify specific target symptoms for each patient and outline the rationale for addressing individuals’ target symptoms using theorized MBI mechanisms of action
• Obtain and document informed consent, including the rationale for, and mechanisms of, MBIs, and describe possible adverse effects
• Address common misconceptions about mindfulness that can become barriers to practice, such as expectation of specific outcomes (e.g., relaxation)
*Medical expert: Plan and perform therapies for the purpose of management*
• Guide MBI-specific mindfulness practices, languaging the instructions to integrate essential elements of practice, such as attentional placement, noting of specific characteristics of objects of attention, and attitudinal underpinnings
• Draw on personal mindfulness practice to exemplify present moment focus and attitudinal underpinnings of mindfulness practice (e.g., receptivity, equanimity, metacognitive awareness) through behavior and verbal and non-verbal communication, utilizing these processes to inform management of the needs of individuals and of the group
• Inquire on MBI-specific mindfulness practices, using an experiential focus to explore the direct experience of practice, reflect on this experience and apply learnings to daily life (i.e., the three layers of inquiry)
• Utilize participants’ descriptions of mindfulness practice during inquiry to inform pacing and presentation of session content in guided practices and discussion
• Understand the integration of mindfulness techniques with cognitive-behavioral techniques, including psychoeducation and behavioral activation
• Foster the recognition and development of metacognitive awareness, guiding participants to practice meta-awareness, disidentification from internal experience, and reduced reactivity to thought content
• Discern between psychiatric symptoms and the arising of mental phenomena associated with meditation “side effects”
• Recognize when to seek supervision from a senior MBI teacher regarding occurrences beyond the limits of one’s expertise, such as management of specific MBI “side effects”
• Contribute to continuous quality improvement of MBIs and attention to patient safety
• Engage in learning and improvement through regular supervision and other means of reflecting on and assessing MBI facilitation skills
*Communicator*
• While embodying mindfulness skills, demonstrate ability to establish, repair when necessary, and maintain therapeutic alliance
*Collaborator*
• Recognize that MBIs are brief treatments in the context of chronic illnesses and negotiate overlapping and shared care responsibilities with clinical colleagues
*Health advocate*
• Facilitate MBIs with awareness of their role for self-management, relapse prevention, and maintaining wellness within and beyond the clinical environment
*Scholar*
• Maintain and expand knowledge and skill base through academic and clinically oriented training materials and regular supervision
• Use assessment and feedback, including from peers and mentors, to inform a professional enhancement plan for ongoing MBI learning
*(c) Advanced expertise–leader stage (self-chosen senior psychiatrists)*
Entrustable Professional Activity (EPA): support effective MBI delivery and integration into healthcare systems, with attention to continuous quality improvement and scholarship (assumes core and AE-therapist milestones are met)
*Medical expert*
• Consult on challenging or unusual clinical situations
• Teach MBI fundamental principles related to the Core and AE-Therapist EPAs
• Supervise AE-Therapists, with fidelity to MBI principles
• Develop modified mindfulness interventions for special patient populations
*Leader*
• Lead quality improvement initiatives related to MBI provision
• Develop and implement MBI delivery models that improve care, value, and efficiency
*Health advocate*
• Respond to the health needs of the population by supporting, planning, and leading program development for MBIs, given their role in self-management, relapse prevention, and maintaining wellness
*Scholar*
• Participate in research efforts, discussing and disseminating research findings, with an understanding of the scientific principles related to MBI theory and practice
• Advance knowledge and skill base through academic and clinical teaching and scholarship, including interdisciplinary collaboration
• Use assessment and feedback to reflect on fulfillment of CanMEDS roles and inform a professional enhancement plan for ongoing MBI learning
*Professional*
• Contribute to the development of standards of competency and ethical codes governing MBI provision
• Provide mentorship to colleagues, exploring challenges and opportunities in MBI provision and inquiring on the mentee’s personal mindfulness practice

Specifically, clinicians need to be aware of potential contraindications to intense practices in MBCT, such as the intolerance of affect associated with borderline personality disorder. These patients are better served by interventions such as Dialectical Behaviour Therapy, in which mindfulness practices are intentionally brief and carefully designed to gradually progress exposure to bodily sensations [21]. In addition, clinicians must consider the suitability of MBI providers, recognizing the lack of standardization among non-certified MBI clinicians and the implications for MBI delivery, safety, and efficacy.

To appropriately inform patients when prescribing MBIs, psychiatrists need to master several knowledge-based milestones: familiarity with a clinically applicable definition of mindfulness, such as the alert, receptive, and equanimous observation of moment-to-moment experience [22], as well as awareness of the mechanisms by which mindfulness leads to symptom improvement, including increased interoceptive awareness, affect tolerance, attention regulation, and the development of metacognitive awareness [23]. They must be able to address common misconceptions that can be barriers to participation, such as mindfulness misconstrued as religious practice, or as detachment from emotions or emptying the mind of thoughts. They may need to clarify the common misunderstanding that mindfulness favors acceptance over taking action. In actuality, mindfulness is a platform for change: some mindfulness-based treatments, such as MiCBT, require patients to identify specific maladaptive behaviors (e.g., those that stem from avoidance) and to define measurable outcomes that indicate their successful resolution (e.g., socializing three times a week in previously avoided situations), thus targeting specific symptoms and functioning [4].

### Advanced Expertise-Therapist Stage of Competency

We propose an AE-Therapist EPA of delivering MBIs with fidelity to key aspects of mindfulness-based teaching. Foremost among the milestones (Table 1b) is an experiential understanding of mindfulness, as it is the clinician’s own experiences with meditation, combined with an understanding of the psychological framework underlying MBIs, that allows for optimal structuring and languaging of practice instructions and inquiry [24]. For example, the development of equanimity, defined as bringing an equal interest to each moment of subjective experience, irrespective of its implicit affective valence (pleasant, unpleasant, or neutral), plays a key role in symptom reduction [25, 26]. Given that developing equanimity includes repeatedly returning attention to unpleasant, unwanted sensations, or pleasant, highly desirable sensations, without enacting even subtle forms of avoidance or attachment, it is nearly impossible for a clinician to guide participants through these practices without having repeatedly worked equanimously with a variety of sensory experiences and emotional states in their own mindfulness practice.

One of the more challenging AE-Therapist milestones is acquiring the skill of inquiry, the interactive investigation of mindfulness practice. During inquiry, the clinician begins by supporting an observational stance through exploration of the patient’s direct experience of meditation, with emphasis on bodily sensations. Attention is drawn to present-moment experience, rather than narratives of the past or forecasts of the future. In the second stage of inquiry, the clinician investigates mental processes that follow from direct experience, such as habitual reactions of attachment or aversion to pleasant and unpleasant sensations. Finally, in the third stage, the clinician assists patients in linking the recognition of these habitual reactions to their role in propagating symptoms, such as panic or avoidance of intimacy. AE-Therapists avoid being formulaic with inquiry but rather conduct it as a relational mindfulness practice [27].

Through mindfulness practice and inquiry, patients develop metacognitive awareness, the interrelated processes of meta-awareness, disidentification from internal experience, and reduced reactivity to thought content [28]. Metacognitive awareness has been shown to contribute to positive outcomes by mediating reduction in anxiety and depressive symptoms [29]. MBI clinicians support participants in relating to thinking as a process (e.g., “I am having thoughts about tomorrow”) instead of identifying with the content of thoughts (e.g., “Tomorrow is going to be a busy day”). Meta-awareness promotes disidentification, allowing patients to relate to experience as transient and arising independently of self-narrative (e.g., “A feeling of happiness is present” rather than “I am happy”). In turn, meta-awareness and disidentification aid the ability to notice thoughts without reacting in habitual ways that perpetuate depressive or anxious states. Participants are then better able to disengage from mental proliferations typical of psychiatric syndromes and to recognize capacity for choice in response to internal and external experiences, particularly those that could precipitate relapse.

Despite the popular view that MBIs are solely benign or beneficial, challenging “side effects” can be encountered [30]. These can be divided into two broad categories [31]. The first includes states that can usually be addressed with guidance by skilled teachers, such as transient dissociative states and increased anxiety. The second, while less common, are effects that may persist during daily life, outside of formal meditation practices. These can include destabilizing symptoms consistent with psychiatric syndromes such as depression, mania, prolonged derealization, psychosis, and suicidality, several of which require swift clinical management. In the traditions from which MBIs are derived, the occurrence of these latter effects is anticipated and proactively managed [24]. The recognition of their potential occurrence has led to the development of training modules for MBSR and MBCT clinicians to include in formal training programs (Britton, 2016 August 30, personal communication) covering adverse effects, multiple interpretive frameworks, and empirically based management strategies. A questionnaire is also under development to aid clinicians in the early detection, corrective instruction, and clinical management of these adverse effects (Britton, 2016 August 30, personal communication). Therefore, clinicians at the AE-Therapist stage must be able to recognize both categories of side effects and provide appropriate psychological and pharmacotherapeutic treatment. Competency includes recognition of the need to consult with AE-Leader clinicians, who can assist with contextualization of experiences for participants and advise on modifying MBI practice instructions.

### Advanced Expertise-Leader Stage of Competency

The AE-Leader EPA involves providing mentorship, supervision and teaching to MBI therapists, and leading integration of MBIs within healthcare systems (Table 1c). These clinicians draw on well-established personal mindfulness practices and in-depth knowledge of meditation phenomena to provide clinical consultation for exigent cases. AE-Leaders also direct systemic quality improvement initiatives, trialling, and assessing optimal means for supporting MBIs as integrated components in stepped-care models for chronic and recurrent mental illness. This involves advocating for the role of MBIs as self-management strategies in systems that have traditionally focused on acute care. AE-Leaders may have the opportunity to support and implement novel MBI delivery models, such as online platforms, in order to improve care and efficiency. AE-Leaders contribute to refining standards of competency, in collaboration with others in the international MBI community, as clinical applications of mindfulness and understanding of underlying mechanisms evolve.

## Implementation of MBI Training

The processes that specialty postgraduate programs will utilize to implement the Competence by Design framework of the Royal College of Physicians and Surgeons of Canada are under development. We describe possible training pathways for successful performance of the three MBI EPAs, providing examples of the breadth of teaching formats, as well as possible challenges in implementation.

### Training Experiences for Core Stage

There is a requisite amount of both didactic and experiential information for clinicians to assimilate to support successful execution of the Core EPA. Additional learning will occur under supervision as residents incorporate MBIs into treatment plans. The milestones (Table 1a) outline required curricular content, such as knowledge of the psychological framework underlying MBIs, and associated mechanisms of symptom reduction. This content could be taught by means of online case-based modules, accessed in a manner synchronized with the learner’s needs. Ideally, comprehensive content could be developed by MBI experts and distributed online nationally. Such non-traditional approaches could assist psychotherapy curriculum coordinators in ensuring high quality and standardized competency for psychiatrists prescribing MBIs.

We posit that online learning alone, however, will not be adequate for acquiring those Core milestones that require an experiential aspect. Seminars led by teachers with AE-leader qualifications are recommended, and these could be video-conferenced from other sites for postgraduate programs without qualified faculty. We strongly recommend that the experiential component link theoretical teaching of the psychological frameworks underlying MBIs with mindfulness practice itself. This can be achieved, for example, by seminars where residents are guided in mindful observation of pleasant and unpleasant sensations and the associated experiences of attachment and aversion. Experiential exposure through seminars supports other medical expert milestones, such as awareness of when personal values or biases may be influencing under- or over-prescription of MBIs. In addition, some programs may consider more extensive mindfulness training for residents by means of a longitudinal course or supported meditation opportunities as part of addressing the well-being component of the CanMEDS professional role.

### Training Experiences for Advanced Expertise-Therapist Stage

We propose that the following training experiences will promote the acquisition of competencies for delivering MBIs:

1. Develop and sustain a personal mindfulness practice that includes formal sitting meditation.
2. Participate in an MBI clinical group for the duration of the group (e.g., 8 sessions). This allows trainees to appreciate the interplay of personal practice, CBT concepts, and group phenomena on an experiential level.
3. Complete a training program that includes theory, experiential practice, and training in inquiry. Formal training should optimally include simulated clinical situations that allow for immediate supervision (e.g., teach-backs or role plays), as well as case conceptualization informed by theoretical frameworks.
4. Co-facilitate three or more manualized MBI groups and/or provide one-on-one manualized MBI to six or more individual patients, with weekly supervision.

These recommendations are consistent with the minimum training guidelines outlined in MBI certification programs and will equip psychiatrists with an MBI skillset that ensures integrity of therapy delivery and promotes shared understanding with certified MBI allied health professionals [13–15]. Where participation in a clinical MBI group is not available (item 2, above), online MBI programs, such as Mindful Noggin (MBCT) or Sounds True (MBSR), offer initial exposure to MBI content and support the development of personal practice.

For residents, training to facilitate MBCT (item 3, above) could be integrated by means of electives, distance-learning training programs, or educational leaves to attend residential components of MBI certification programs (e.g., MBSR, MBCT, MiCBT). For example, the University of California San Diego Mindfulness-Based Professional Training Institute offers 5-day residential trainings that can be completed individually or as part of certification programs [32]. Comprehensive MiCBT training can be obtained through an online certification program, removing geographical limitations; trainees can also access ongoing online MiCBT group supervision after completion of the 3-month online course [33]. With regards to clinical supervision (item 4, above) where local AE-Leader supervisors are not available, trainees can access regular supervision through external MBI training programs that include online mentorship with advanced teachers or through supervision with AE-Leaders at other psychiatry programs [32].

### Training Experiences for Advanced Expertise-Leader Stage

AE-Leader competency is developed through focused work with MBI delivery, supervision, academic writing and teaching, and collaboration with peers. While these pursuits are largely self-directed, they can be supported by establishing mentorship relationships, both with clinical and contemplative practice mentors. Ongoing personal practice continues to form the foundation for development of milestones, and extended practice, such as annual 5–7-day silent meditation retreats, is recommended. A cadre of Canadian leaders needs to be developed to assist with educational product development and to establish accessible, psychiatric-specific supervision, and Continuing Professional Development resources.

In summary, in the context of the upcoming transformational redesign of Canadian postgraduate training to a Competence by Design framework, we propose competency-based guidelines for training in mindfulness-based interventions. MBIs are an emerging mental health treatment, and thus invite explication of competencies and incorporation of novel training methods into psychiatry postgraduate training programs. Ideally, feedback mechanisms will be built into the new assessment framework, allowing effectiveness of competency guidelines, such as those presented, to be iteratively assessed and improved.

We have structured the guidelines around three specific EPAs and recommend that all psychiatry trainees develop competency for the Core of Discipline EPA of assessing suitability for, and prescribing, MBIs. A subset of residents and psychiatrists in the Advanced Expertise stage of the competency continuum may elect to develop proficiency with the EPA of MBI delivery, and we have defined detailed milestones that can be utilized to develop a precise and comprehensive skillset that has personal meditation practice as its core. The advanced training encourages innovative formats that may include brief residential training and online group and individual supervision. Thus, even clinicians in under-resourced areas can access MBI leaders, who have a vital role in training, mentoring, and assuring quality and scholarship. As learning methods evolve, Canada-wide MBI competencies have the potential to standardize training and ensure a baseline of MBI knowledge within the Canadian psychiatric community.
